# Prevalence and antimicrobial resistance of *Streptococcus suis* isolated from local pig breeds in Jiangxi Province, China

**DOI:** 10.3389/fvets.2025.1582461

**Published:** 2025-08-04

**Authors:** Mei-Fang Tan, Chen-Long Liu, Quan-Yong Zhou, Jia-Zhen Wei, Jia-Wei Hong, Ming-Chun Wan, Feng-Lin Zhang, Hua-Yuan Ji

**Affiliations:** ^1^Institute of Animal Husbandry and Veterinary Science, Jiangxi Academy of Agricultural Sciences, Nanchang, China; ^2^Department of Animal Science, Jiangxi Biological Vocational College, Nanchang, China

**Keywords:** *Streptococcus suis*, prevalence, antimicrobial resistance, local pig breed, Jiangxi Province

## Abstract

**Introduction:**

Jiangxi Province possesses abundant genetic resources of local pig breeds, whose effective conservation is essential for maintaining biodiversity and sustainable utilization. *Streptococcus suis* is an important zoonotic pathogen that continuously threatens swine production systems and public health globally. This study aimed to investigate the epidemiological characteristics of *S. suis* among local pig breeds in Jiangxi Province.

**Methods:**

An investigation was conducted on the prevalence and antimicrobial resistance profiles of *S. suis* in six local pig breeds from Jiangxi Province, including Gandong black pig, Hang pig, Ganxi two-end-black pig, Dongxiang spotted pig, Yushan black pig, and Binhu black pig.

**Results:**

A total of 340 porcine nasal swabs were collected from six local pig breeds. 208 *S. suis* strains were isolated from 187 samples, with an overall isolation rate of 55.0%. The positive isolation rates of the six local breeds were 58.0, 80.0, 71.7, 51.7, 13.3, and 60.0%, respectively. Antimicrobial susceptibility testing revealed that >98% of isolates were susceptible to carbapenems (meropenem and imipenem), followed by doxycycline (71.6%), ceftiofur (65.9%), spectinomycin (64.4%), and amoxicillin (55.8%). High resistance rates were observed for tilmicosin (96.6%), sulfadiazine (92.8%), colistin (89.9%), apramycin (88.9%), chlortetracycline (87.5%), tiamulin (83.2%), and kanamycin (79.8%). 100% of isolates exhibited multidrug resistance, with significant variations in resistance patterns among breeds. Genotypic analysis identified *ermB* (macrolides), *tetL* (tetracyclines), and *Sul2* (sulfonamides) as the predominant resistance determinants.

**Discussion:**

Multidrug-resistant *S. suis* strains have become widespread among local pig breeds. This study could provide evidence-based guidance for developing effective prevention and control strategies against *S. suis* infections and protecting valuable genetic resources of indigenous pig breeds.

## Introduction

Through thousands of years of domestication and natural selection, Chinese local pig breeds have developed distinct genetic characteristics, contributing to global genetic diversity and the sustainable development of the pig industry (1). Compared with commercial Western pig breeds, Chinese local pigs typically exhibit superior resistance to coarse feed and environmental stresses (2). Jiangxi Province is home to one of the largest populations of native pig breeds in China, accounting for approximately one-tenth of the country’s local pig genetic resources (3). However, over the past decade, many of these local breeds have faced near extinction due to multiple factors, including competition from commercial Western pig breeds, small population sizes, and threats from epidemic diseases (2). Effective conservation of these breeds is essential for their preservation and future utilization.

*Streptococcus suis* is a Gram-positive bacterial pathogen that causes porcine respiratory tract infections and severe invasive diseases, including meningitis, septicemia, and arthritis (4). As an important swine pathogen, *S. suis* is responsible for substantial economic losses in the global swine industry (5). Moreover, it has emerged as an important zoonotic agent, with frequent cases of human transmission, particularly in Asia (5). The major outbreaks of *S. suis* in China in 1998 and 2005 brought global attention to this pathogen (6). Continued reports of human cases across 22 Chinese provinces further emphasize the persistent threat of this zoonotic infection (7). Consequently, *S. suis* infections in pig populations threaten not only agricultural sustainability but also public health (8). Although antimicrobial prophylaxis and therapy have been successful in controlling these infections, the emergence and spread of antibiotic-resistant *S. suis* strains elevate the risks of treatment failure in both pigs and humans (9). Therefore, monitoring antimicrobial resistance (AMR) profiles of *S. suis* in specific regions is essential for optimizing effective antimicrobial therapies and tracking the development of bacterial drug resistance (10).

In recent years, antimicrobial resistance (AMR) phenotypes and genotypes of *S. suis* isolates from clinically healthy commercial three-way crossbred pigs (2017–2019) in Jiangxi Province, China, have been characterized (11). However, data on *S. suis* in local pig breeds within this Province remain unknown. In the present study, *S. suis* strains were isolated from nasal cavities of local breed piglets and evaluated their antimicrobial susceptibility profiles. These findings may provide evidence-based guidance for *S. suis* infection prevention and control, while contributing to the conservation of porcine germplasm resources in China.

## Materials and methods

### Clinical sample collection

**Figure 1:**
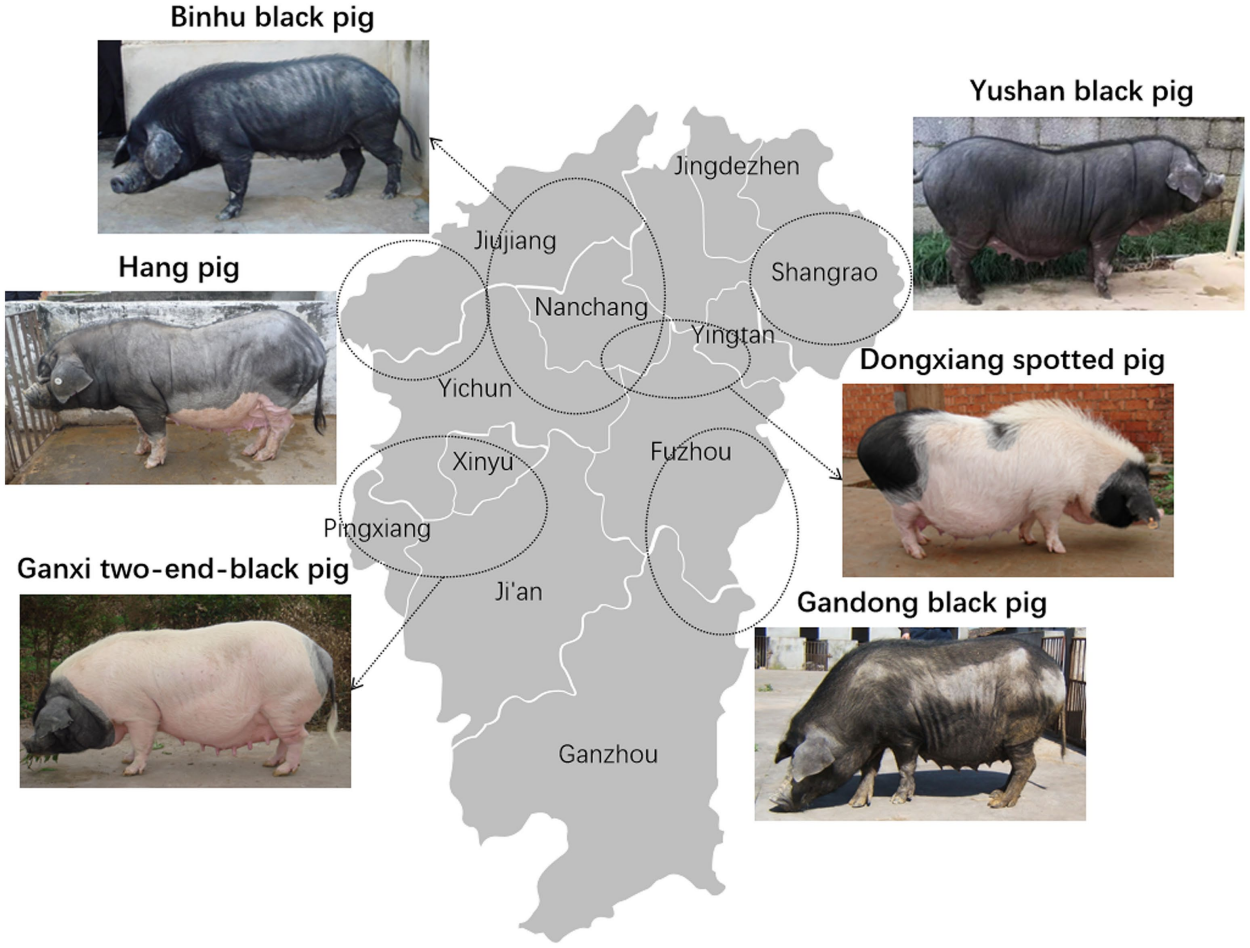
Distribution of specific local pig breeds in Jiangxi Province.

From March 2023 to September 2024, 340 nasal swab samples were collected from clinically healthy weaned piglets (aged 30–76 days) representing six local breeds in Jiangxi Province, China: Gandong black pig, Hang pig, Ganxi two-end-black pig, Dongxiang spotted pig, Yushan black pig, and Binhu black pig (Figure 1). Samples were obtained from two distinct batches per breed with sterile cotton swabs (Table 1). Swabs were then stored in 1.5 mL of transport buffer within bacterial sampling tubes (Youkang Biotechnology, Beijing, China) and transported to the laboratory at a low temperature.

**Table 1:** Positive carrier rates of *Streptococcus suis* in local pig breeds.

Local pig breed	Sampling time	No. of nasal swabs	Positive rate	Average positive rate	No. of bacterial isolates
Gandong black pig	March 2023	28	20/28 (71.4%)	29/50 (58.0%)	38
	July 2023	22	9/22 (40.9%)		9
Hang pig	August 2023	20	11/20 (55.0%)	40/50 (80.0%)	8
	April 2024	30	29/30 (96.7%)		33
Ganxi two-end-black pig	April 2024	30	20/30 (66.7%)	43/60 (71.7%)	20
	May 2024	30	23/30 (76.7%)		21
Dongxiang spotted pig	April 2024	30	18/30 (60.0%)	31/60 (51.7%)	18
	September 2024	30	13/30 (43.3%)		14
Yushan black pig	June 2024	30	4/30 (13.3%)	8/60 (13.3%)	4
	September 2024	30	4/30 (13.3%)		4
Binhu black pig	August 2024	30	8/30 (26.7%)	36/60 (60.0%)	11
	September 2024	30	28/30 (93.3%)		28
Total		340		187/340 (55.0%)	208

### Bacterial isolation and identification

The liquid samples were cultured aerobically on Columbia agar (Hopebio, Qingdao, China) plates supplemented with 5% sterile defibrinated sheep blood (Hopebio) at 37°C for 24 h. Alpha-hemolytic colonies exhibiting typical morphology were subjected to Gram staining. Suspected *S. suis* colonies were then analyzed by PCR targeting the 16S rRNA gene using primer pair 27F/1492R (Table S1). The amplification used the following cycling parameters: one cycle of 98°C for 8 min, followed by 30 cycles of 95°C for 30 s, 57°C for 30 s, and 72°C for 1 min. Amplification products were sent to Sangon Biotech (Shanghai) Co., Ltd. for sequencing. The resulting sequences were analyzed using NCBI’s Nucleotide BLAST with default parameters.

### Antibiotic drugs

A total of 20 commercially available antimicrobial agents for veterinary and human use were prepared for antimicrobial susceptibility testing, including: penicillin [amoxicillin (AMX) and ampicillin (AMP)], cephalosporins [ceftiofur (FUR)], tetracyclines [doxycycline (DX) and chlortetracycline (CT)], phenicols [florfenicol (FFC)], macrolides [erythromycin (E) and tilmicosin (TIL)], polypeptides [colistin sulfate (CS)], sulfonamides [sulfadiazine (SUZ)], quinolones [enrofloxacin (ENR) and ciprofloxacin (CIP)], diterpenes [tiamulin (T)], aminoglycosides [neomycin (N), spectinomycin (SPT), gentamicin (GM), kanamycin (K), and apramycin (AP)], and carbapenems [imipenem (IMI) and meropenem (MRP)]. Antibiotic discs containing CT were obtained from HiMedia Laboratories (Mumbai, India), while all other discs were sourced from Liofilchem S.r.l. (Roseto degli Abruzzi, Italy). The drug concentrations in each disc are detailed in Table S2.

### Antimicrobial susceptibility assay

The antimicrobial susceptibility of all isolates was determined using the Kirby-Bauer disk diffusion method on Mueller-Hinton (MH) agar (Hopebio) supplemented with 5% newborn bovine serum (Sijiqing, Hangzhou, China), following the standardized protocol recommended by the Clinical and Laboratory Standards Institute (CLSI) (12). Briefly, *S. suis* strains were cultured on MH agar plates at 37°C under 5% CO_2_ for 12 h. Selected colonies were suspended in normal saline and adjusted to a 0.5 McFarland standard. Bacterial suspensions were uniformly spread on MH plates using sterile cotton swabs. Antibiotic discs were aseptically placed using a disc dispenser, and plates were incubated at 37°C in 5% CO_2_ for 18 h. *Streptococcus pneumoniae* ATCC 49619 served as the quality control strain. Isolates were categorized as susceptible, intermediate, or resistant based on established criteria (Supplementary Table S2). For three carbapenem-resistant *S. suis* strains, minimum inhibitory concentrations (MICs) of imipenem and meropenem were determined using MIC Test Strips (Liofilchem).

Notably, since species-specific CLSI breakpoints for *S. suis* are currently unavailable, the standards established for *S. pneumoniae* (non-meningitis isolates) were adopted in this study.

### Identification of antibiotic resistance genes

All *S. suis* isolates were cultured overnight at 37°C in tryptone soy broth (Hopebio) supplemented with 5% newborn bovine serum. Genomic DNA was extracted using bacterial DNA kits (Cwbiotech, Beijing, China) following the manufacturer’s instructions. PCR amplification was performed to detect resistance genes for macrolides (*erm*A, *erm*B, and *mef*A) (13), tetracyclines (*tet*L, *tet*O, *tet*K, and *tet*M) (14), aminoglycosides [*aph(3′)*], and sulfonamides (*Sul1*, *Sul2*, and *Sul3*) (15). The 11 primer sets, along with their expected amplicon sizes and annealing temperatures, are listed in Supplementary Table S1. The PCR cycling conditions were as follows: 1 cycle at 95°C for 5 min, followed by 30 cycles of 95°C for 30 s, Tm for 30 s, and 72°C for 1 min. PCR products were analyzed using electrophoresis on 1.0% (wt/vol) agarose gels.

### Statistical analysis

Data visualization was conducted with GraphPad Prism 8.0 software (San Diego, USA). Statistical analysis was performed using IBM SPSS Statistics 25.0 (Amonk, USA). Intergroup comparisons were analyzed by Chi-square tests with false discovery rate (FDR) correction for multiple comparisons in SPSS. A two-tailed FDR-adjusted *p*-value <0.05 was considered statistically significant.

### Ethics statement

All sampling procedures were approved by the Animal Ethics Committee of the Institute of Animal Husbandry and Veterinary Medicine, Jiangxi Academy of Agricultural Science (2020-JXAAS-XM-01).

## Results

### Prevalence of *S. suis* in local pig breeds

According to current Chinese agricultural industry standards, the Gandong black pig, Hang pig, Dongxiang spotted pig, and Binhu black pig are classified as endangered breeds; the Yushan black pig as a vulnerable protected breed; and the Ganxi two-end-black pig as a low-risk breed (Figure 1) (3). From March 2023 to September 2024, 340 porcine nasal swabs from six local pig breeds were obtained for *S. suis* isolation. From each sample, five suspected bacterial colonies were subjected to Gram staining and PCR identification. Results revealed an overall *S. suis* carrier rate of 55.0% (187/340; Table 1), with breed-specific rates of 58.0% (29/50), 80.0% (40/50), 71.7% (43/60), 51.7% (31/60), 13.3% (8/60), and 60.0% (36/60) (*χ*^2^ = 62.05, *p* < 0.001), demonstrating statistically significant differences among breeds. A total of 208 *S. suis* strains were stored and used for subsequent antimicrobial susceptibility assays.

### Antimicrobial susceptibility profiles

**Figure 2:**
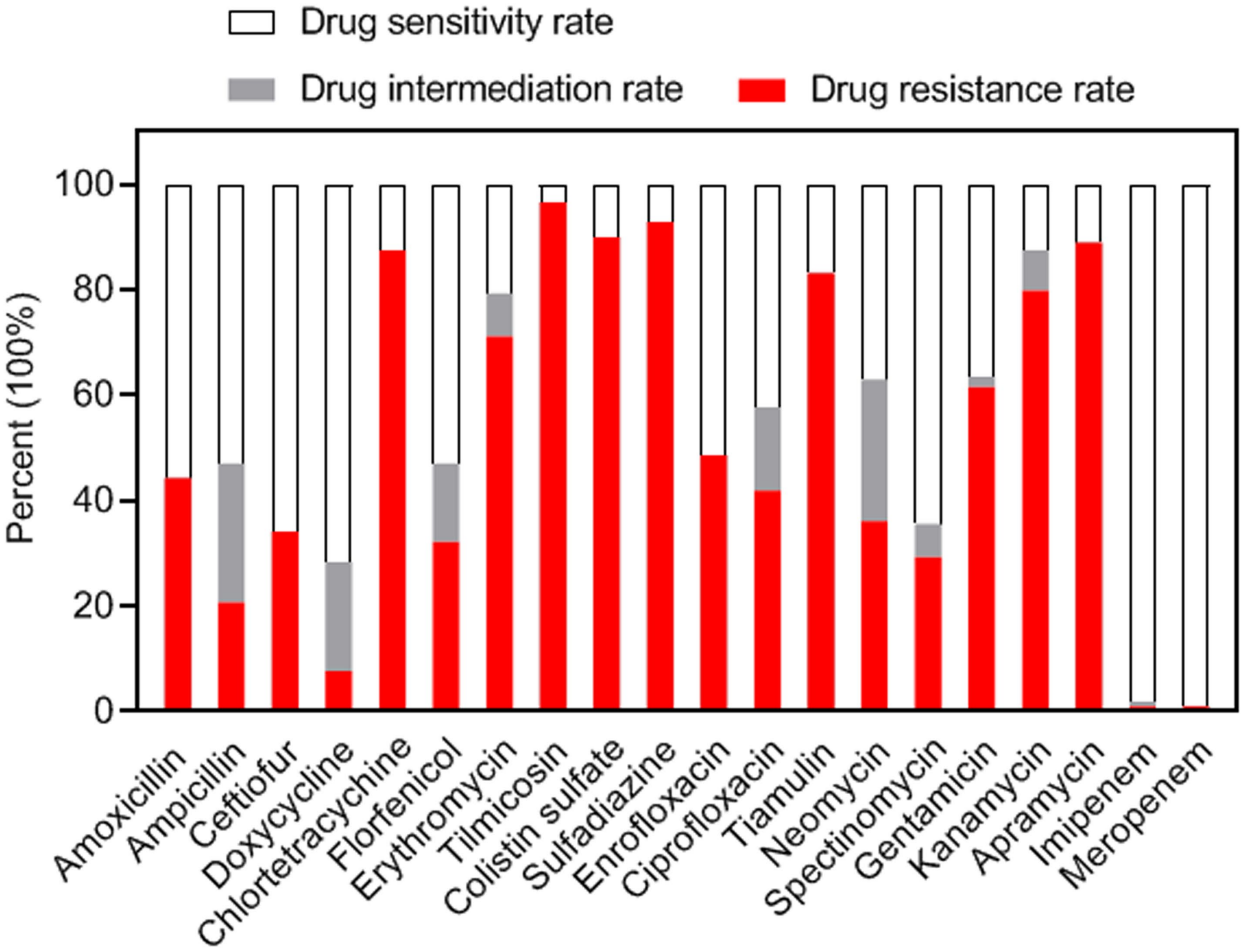
Antimicrobial resistance profiles of the *S. suis* isolates (*n* = 208).

The antimicrobial susceptibility profiles of 208 *S. suis* strains were assessed using the disc diffusion method with 20 antimicrobial agents. Inhibition zone diameters for all tested antibiotics are presented in Supplementary Table S3. Results demonstrated high susceptibility rates (>98%) to MRP (99.0%) and IMI (98.1%), followed by DX (71.6%), FUR (65.9%), SPT (64.4%), and AMX (55.8%) (Figure 2). Conversely, high resistance frequencies were observed to TIL (96.6%), SUZ (92.8%), CS (89.9%), AP (88.9%), CT (87.5%), T (83.2%), and K (79.8%) (Figure 2). The highest intermediate resistance level was observed for N (26.9%).

Notably, three carbapenem-resistant strains demonstrating resistance to imipenem and/or meropenem were identified. Results of additional MIC testing confirmed these strains exhibited resistance to imipenem and/or meropenem (Supplementary Table S4). The emergence of carbapenem-resistant strains warrants particular concern given the limited therapeutic options available for possible infections.

### Drug sensitivity of *S. suis* from different local pig breeds

*S. suis* strains exhibited similarly high resistance rates to imipenem (IMI) and meropenem (MRP), while susceptibility to other antibiotics varied significantly across isolates from the six breeds (Table 2). For instance, AMX susceptibility rates were 78.7% (37/47) in Gandong black pigs, 17.1% (7/41) in Hang pigs, 21.3% (33/41) in Ganxi two-end-black pigs, 93.8% (30/32) in Dongxiang spotted pigs, 75.0% (6/8) in Yushan black pigs, and 7.7% (3/39) in Binhu black pigs (*p* < 0.001). Similarly, enrofloxacin (ENR) resistance rates were 74.5% (35/47), 29.3% (12/41), 48.8% (20/41), 100.0% (32/32), 75.0% (6/8), and 5.1% (2/39), respectively (*p* < 0.001).

**Table 2:** Drug sensitivity rates of *Streptococcus suis* isolated from different pig breeds.

Antimicrobial category	Antimicrobial drug	Local pig breed^a^						*p* value^b^
		Gandong black pig (*n* = 47)	Hang pig (*n* = 41)	Ganxi two-end-black pig (*n* = 41)	Dongxiang spotted pig (*n* = 32)	Yushan black pig (*n* = 8)	Binhu black pig (*n* = 39)	
Penicillin	Amoxicillin	78.7% (37)	17.1% (7)	21.3% (33)	93.8% (30)	75.0% (6)	7.7% (3)	*P* < 0.001
	Ampicillin	72.3% (34)	17.1% (7)	73.2% (30)	93.8% (30)	87.5% (7)	2.6% (1)	*P* < 0.001
Cephalosporin	Ceftiofur	70.2% (33)	53.7% (22)	58.5% (24)	100.0% (32)	75.0% (6)	51.3% (20)	*p =* 0.002
Tetracycline	Doxycycline	61.7% (29)	73.2% (30)	51.2% (21)	93.8% (30)	75.0% (6)	84.7% (33)	*p =* 0.006
	Chlortetracycline	2.1% (1)	2.4% (1)	2.4% (1)	3.1% (1)	–	56.4% (22)	*p* = 0.03
Chloramphenicol	Florfenicol	83.0% (39)	2.4% (1)	31.7% (13)	68.8% (22)	100.0% (8)	66.7% (26)	*p* < 0.001
Macrolide	Erythromycin	2.1% (1)	7.3% (3)	17.1% (7)	3.1% (1)	62.5% (5)	66.7% (26)	*p* = 0.002
	Tilmicosin	2.1% (1)	–	–	–	–	15.4% (6)	*p* = 0.003
Polypeptide	Colistin sulfate	–	22.0% (9)	7.3% (3)	3.1% (1)	12.5% (1)	17.9% (7)	*p* = 0.001
Sulfonamide	Sulfadiazine	–	–	9.8% (4)	18.8% (6)	25.0% (2)	7.7% (3)	*p* = 0.001
Quinolone	Enrofloxacin	74.5% (35)	29.3% (12)	48.8% (20)	100.0% (32)	75.0% (6)	5.1% (2)	*p* < 0.001
	Ciprofloxacin	51.1% (24)	19.5% (8)	51.2% (21)	90.6% (29)	62.5% (5)	2.6% (1)	*p* < 0.001
Diterpene	Tiamulin	2.1% (1)	4.9% (2)	26.8% (11)	56.3% (18)	37.5% (3)	–	*p* = 0.002
Aminoglycoside	Neomycin	53.2% (25)	39.0% (16)	14.6% (6)	25.0% (8)	25.0% (2)	51.3% (20)	*p* = 0.014
	Spectinomycin	83.0% (39)	39.0% (16)	75.6% (31)	68.8% (22)	62.5% (5)	53.8% (21)	*p* < 0.001
	Gentamicin	2.1% (1)	14.6% (6)	78.0% (32)	31.3% (10)	62.5% (5)	56.4% (22)	*p* = 0.006
	Kanamycin	–	2.4% (1)	12.2% (5)	28.1% (9)	37.5% (3)	20.5% (8)	*p* = 0.002
	Apramycin	–	2.4% (1)	2.4% (1)	50.0% (16)	–	12.8% (5)	*p =* 0.008
Carbapenem	Imipenem	100.0% (47)	100% (41)	95.1% (39)	100.0% (32)	100.0% (8)	100.0% (39)	NS
	Meropenem	100.0% (47)	97.6% (40)	95.1% (39)	100.0% (32)	100.0% (8)	100.0% (39)	NS

a Dash (−) indicates no strains were observed in this classification.

b NS indicates no significance.

**Figure 3:**
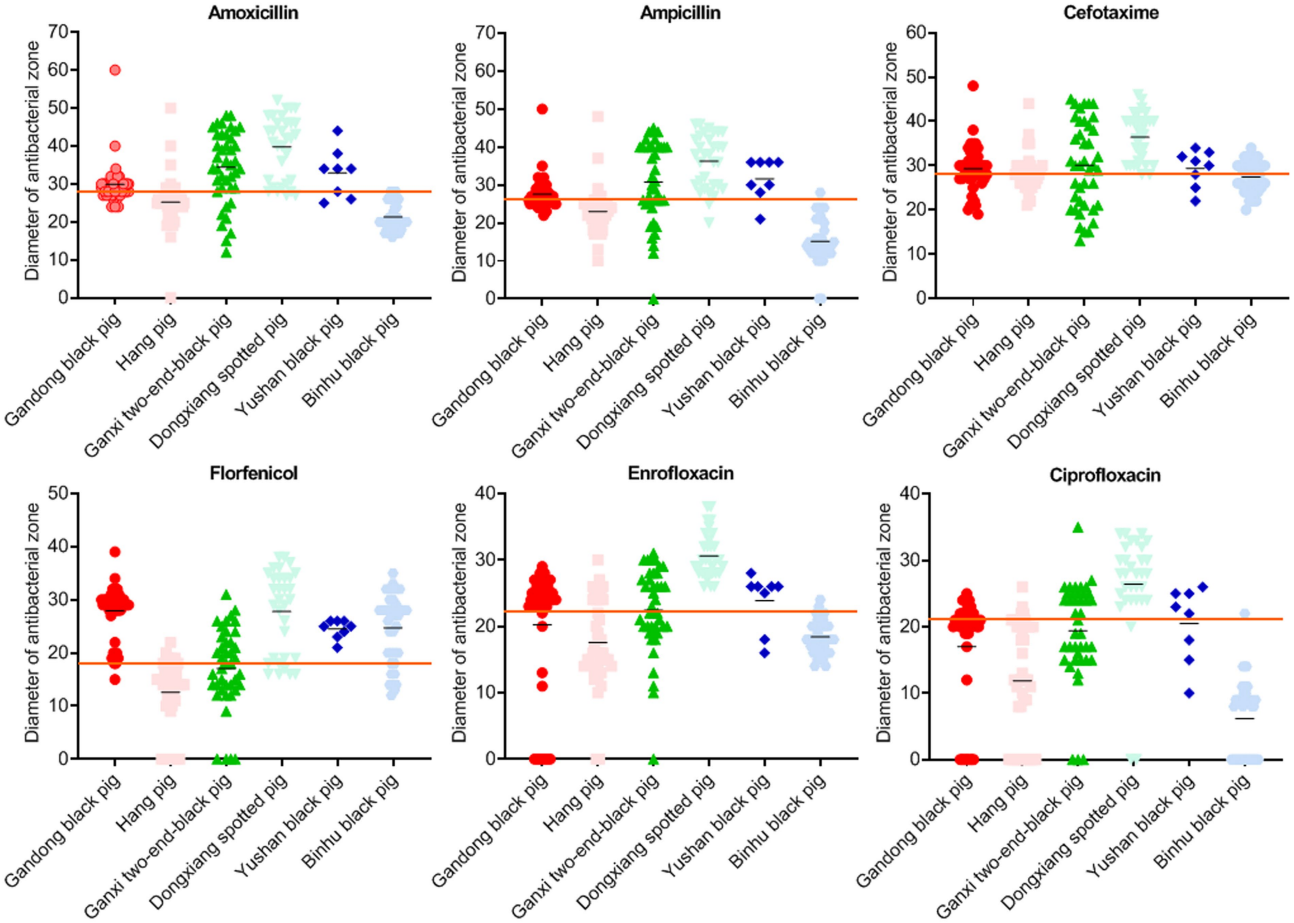
Inhibition zone diameters of bacteria isolated from six local pig breeds against specific drugs. X-axes of scatter diagrams indicate the six local pig breeds. Short black lines represent average values. Long red lines represent sensitivity thresholds of specific antimicrobials.

We further analyzed inhibition zone diameters for six key antibiotics (AMX, AML, FUR, FFC, ENR, and CIP), which are commonly used to control *S. suis* infections in swine production (16). While Hang pig isolates showed high resistance to all six drugs, Dongxiang spotted pig isolates displayed markedly lower resistance (Figure 3). This disparity indicates a significant correlation between bacterial isolation source (i.e., pig breed) and antimicrobial susceptibility profiles.

Except for IMI and MRP, the most sensitive drugs for *S. suis* in Gandong black pigs were FFC (83.0%) and SPT (83.0%), in Hang pigs was DX (73.2%), in Ganxi two-end-black pigs was GM (78.0%), in Dongxiang spotted pigs were FUR (100.0%) and ENR (100.0%), in Yushan black pigs was FFC (100.0%), and in Binhu black pig was DX (84.7%) (Table 2). These findings provide evidence-based guidance for clinical prevention and treatment strategies.

### Multidrug resistance

**Figure 4:**
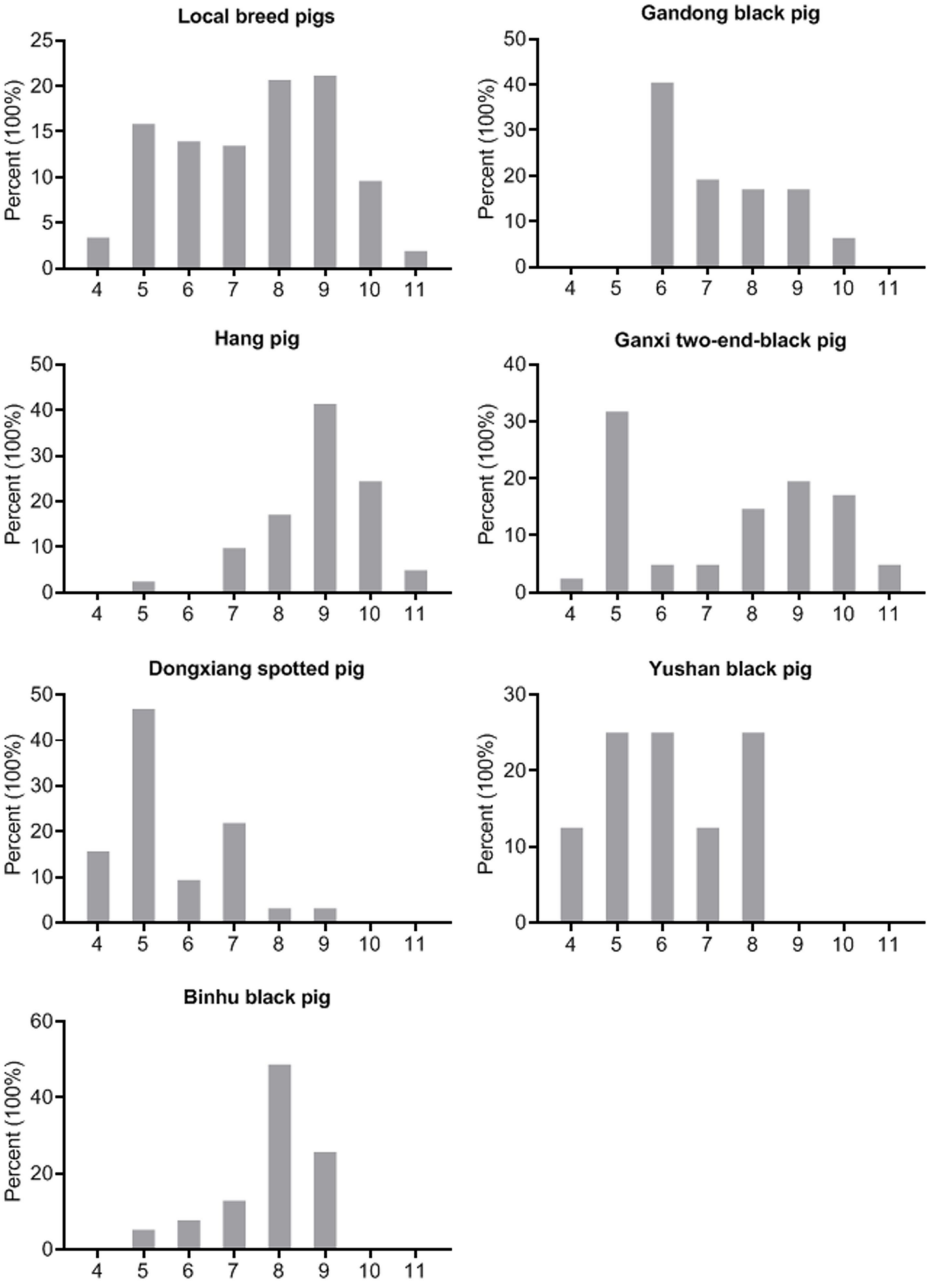
Multidrug resistance of the *S. suis* isolates (*n* = 208). X-axes of seven histograms indicate resistance of the *S. suis* isolates to 4–11 categories of antimicrobials.

The standardized definition of multidrug resistance refers to bacterial isolates exhibiting non-susceptibility to at least three antimicrobial categories (17). All *S. suis* isolates in this study demonstrated resistance to a minimum of four antimicrobial categories (Table S3), with 100.0% classified as multidrug-resistant (Figure 4). In addition, 38.5% (80/208) of the isolates displayed the most prevalent antimicrobial resistance pattern (CT-E-TIL-CS-SUZ-T-GM-AP). Furthermore, 21.2% (44/208) of isolates exhibited resistance to nine antimicrobial drugs, while 20.7% (43/208) showed resistance to eight antimicrobial drugs, representing the predominant resistance profiles observed.

As shown in Figure 4, the predominant multidrug resistance profiles varied among breeds: resistance to six antimicrobial categories in Gandong black pigs (40.4%, 19/47), nine categories in Hang pigs (41.5%, 17/41), five categories in Ganxi two-end-black pigs (31.7%, 13/41), five categories in Dongxiang spotted pigs (46.9%, 15/32), five/six/eight categories in Yushan black pigs (25.0%, 2/8), and eight categories in Binhu black pigs (48.7%, 19/39). These findings demonstrate substantial variation in multidrug resistance patterns of *S. suis* across different indigenous pig breeds.

Moreover, the three carbapenem-resistant strains exhibited extensive drug resistance to all 11 antimicrobial categories tested, with one strain being resistant to all 20 drugs (Table S3).

### Detection of drug resistance genes

**Figure 5:**
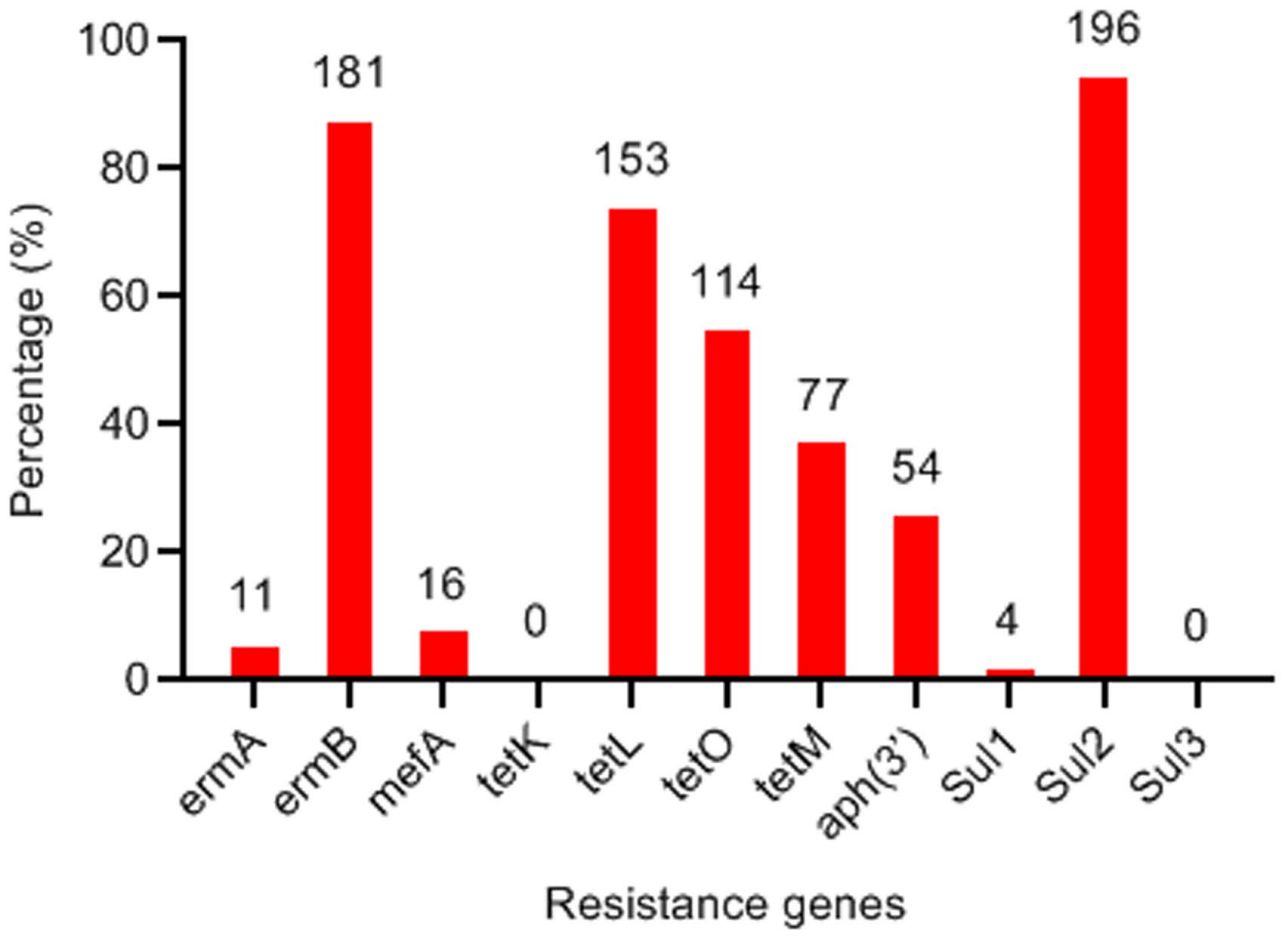
Detection frequency of antimicrobial resistance genes in *Streptococcus suis* isolates (*n* = 208). The numbers on the columns indicate the number of *S. suis* isolates possessing the corresponding resistance genes.

Due to the high frequency of resistance to macrolides, tetracyclines, aminoglycosides, and sulfonamides, all strains were screened for corresponding resistance genes using PCR assays (Figure 5). The most prevalent macrolide resistance gene was *ermB* (87.0%, 181/208), followed by *mefA* (7.69%, 16/208). Among the tetracycline resistance genes, *tetL* (73.6%), *tetO* (54.8%), and *tetM* (37.0%) were detected, whereas *tetK* was absent (0%). The aminoglycoside resistance gene *aph(3′)* was identified in 26% (54/208) of the isolates. Additionally, *Sul2* was the predominant sulfonamide resistance determinant in *S. suis*, present in 94.2% (196/208) of the strains.

## Discussion

Disease prevention and control are critical for protecting the germplasm resources of Chinese local pig breeds. *S. suis* naturally colonizes the upper respiratory tract of healthy pigs and is commonly detected in commercial pig populations across most provinces of China (7). Previous studies indicate that *S. suis* prevalence is highest in smallholder farms, with pigs under 6 months of age being more susceptible to infection than older pigs (18). Additionally, the zoonotic risk posed by *S. suis* remains underappreciated, even after the two largest outbreaks in China in 1998 and 2005, and its prevalence is particularly concerning in regions with intensive pig farming (19). Given this background, we investigated the prevalence and antimicrobial resistance of *S. suis* in six local pig breeds in Jiangxi Province. This study aims to reduce disease risks in the local pig breed production system and provide baseline data to enhance biosecurity measures.

The positive carrier rate of *S. suis* in the six pig breeds ranged from 13.3 to 80.0%, with an average of 58.0%. This rate is higher than both the 34.08% prevalence previously reported in Jiangxi Province’s commercial pigs (11) and the 15.8% prevalence observed in smallholder farms in the Philippines (20). These findings suggest that local pig populations in Jiangxi Province are at high risk of *S. suis* infection.

Antimicrobial drugs have long been proven effective in promoting growth and preventing bacterial infections. However, *S. suis* has rapidly developed antibiotic resistance through horizontal gene transfer (21). Multidrug-resistant *S. suis* strains have been identified across all serotypes, time periods, and geographical regions worldwide (22). The results of antimicrobial susceptibility assays revealed that 100% of the isolates in this study were multidrug-resistant. The human-use-only antimicrobials IMI and MRP remained the most effective agents. However, high resistance rates were observed to commonly used veterinary antimicrobial drugs against *S. suis*, including AMX (44.2%), AMP (47.1%), SUZ (92.8%), E (79.3%), ENR (48.6%), CIP (57.7%), and T (83.2%). These findings raise serious concerns about potential treatment failures in the future.

The positive carrier rate, antimicrobial susceptibility, and multidrug resistance patterns of *S. suis* varied among local pig breeds. The widespread emergence of resistant bacterial strains typically results from antimicrobial selective pressure (23). Consequently, the observed interbreed differences in bacterial characteristics may be attributed to not only porcine genetic factors but also variations in farm management levels and medication strategies. The highest isolation rates of *S. suis* occurred in Hang pigs (80.0%) and Ganxi two-end-black pigs (71.7%). Furthermore, these two populations contained carbapenem-resistant strains that demonstrated multidrug resistance across all tested antimicrobial categories. The emergence of carbapenem-resistant *S. suis* represents a significant public health concern due to its implications for both veterinary and human clinical treatment (24, 25). Carbapenem resistance in *Streptococcus* spp. has been increasingly reported in human clinical cases (26, 27) and diseased pigs (22) in recent years. These findings underscore the need for current *S. suis* control strategies to emphasize both appropriate antimicrobial selection and prudent usage (10). In this study, we have identified the most effective antibiotics against *S. suis* infection for each local pig breed, findings that may help mitigate the impact and transmission of resistant *S. suis* strains.

Resistance to macrolides is primarily mediated by ribosomal methylases encoded by *erm* genes, or less commonly, by macrolide efflux pumps encoded by *mef* genes (13). The *ermB* gene was the most prevalent resistance gene in *S. suis* isolates, observed in both locally bred pigs in this study and commercial pigs (11). Previous studies have demonstrated that *tetO* represents the predominant tetracycline resistance gene in *S. suis* isolates from pigs and humans in Italy, Japan, Korea, and Jiangxi Province (11). In contrast, our results revealed *tetL* as the most prevalent tetracycline resistance gene among *S. suis* isolates from locally bred pigs. Sulfonamide resistance is known to be conferred by *sul* genes (15). The high detection rate of *Sul2* (94.2%) is consistent with the resistance rate of the isolates to SUZ (92.8%).

Notably, the complete information of the other resistance genes and mobile genetic elements in these strains remains uncharacterized. As previously described, mobile genetic elements harboring multiple AMR genes and virulence factors facilitate the horizontal dissemination of AMR determinants across the intra- and inter-species (11). Multidrug-resistant *S. suis* not only poses a direct threat to swine production and public health but also serves as a reservoir for AMR gene transmission to clinically relevant streptococcal pathogens, including *Streptococcus agalactiae*, *Streptococcus pneumoniae*, and *Streptococcus pyogenes* (28). Therefore, further genome sequencing and bioinformatics analysis would provide more detailed information about these *S. suis* strains and would be more helpful for developing targeted prevention strategies and control interventions.

## Conclusion

This study provides the first investigation of *S. suis* carrier rates and detailed antimicrobial resistance profiles among six representative local pig breeds in Jiangxi Province, demonstrating a high prevalence in clinically healthy piglets. Antimicrobial susceptibility assays revealed widespread multidrug resistance among *S. suis* isolates, with significant breed-specific variations in therapeutic efficacy of antimicrobials used for *S. suis* prevention and treatment. These findings highlight the urgent need for coordinated government-farmer collaborations to implement antimicrobial stewardship programs, establish continuous AMR surveillance systems, and advance research into resistance mechanisms to develop novel prevention strategies against *S. suis* infection.

## Data Availability

The original contributions presented in the study are included in the article/Supplementary material, further inquiries can be directed to the corresponding author.
