# Supplementary material for: Prevalence and antimicrobial resistance of Streptococcus suis isolated from local pig breeds in Jiangxi Province, China
Source: Front Vet Sci. 2025 Aug 4;12:1582461. doi: 10.3389/fvets.2025.1582461 (PMC12358291; doi:10.3389/fvets.2025.1582461)
Supplement: Supplementary file 1 [file Table_1.DOCX]

Table S1. PCR primers used in this study.

| Gene targeted | Primer sequence (5’- 3’) | Fragment size | Tm value | Reference |
| --- | --- | --- | --- | --- |
| 16S rDNA | F: AGAGTTTGATCCTGGCTCAG | 1465 bp | 57℃ | ([Frank et al., 2008](#_ENREF_1)) |
|  | R: TACGGTTACCTTGTTACGACTT |  |  |  |
| *erm*A | F: TCTAAAAAGCATGTAAAAGAA | 645 bp | 50℃ | ([Jacobs et al., 2003](#_ENREF_2)) |
|  | R: CTTCGATAGTTTATTAATATTAGT |  |  |  |
| *erm*B | F: GAAAAGGTACTCAACCAAATA | 639 bp | 52℃ |  |
|  | R: AGTAACGGTACTTAAATTGTTTAC |  |  |  |
| *mef*A | F: AGTATCATTAATCACTAGTGC | 348 bp | 52℃ |  |
|  | R: TTCTTCTGGTACTAAAAGTGG |  |  |  |
| *tet*K | F: TATTTTGGCTTTGTATTCTTTCAT | 1159 bp | 52℃ | ([Zhang et al., 2015](#_ENREF_4)) |
|  | R: GCTATACCTGTTCCCTCTGATAA |  |  |  |
| *tet*L | F: ATAAATTGTTTCGGGTCGGTAAT | 1077 bp | 56℃ |  |
|  | R: AACCAGCCAACTAATGACAATG |  |  |  |
| *tet*O | F: AACTTAGGCATTCTGGCTCAC | 519 bp | 58℃ |  |
|  | R: TCCCACTGTTCCATATCGTCA |  |  |  |
| *tet*M | F: GAACTCGAACAAGAGGAAAGC | 740 bp | 56℃ |  |
|  | R: ATGGAAGCCCAGAAAGGAT |  |  |  |
| *aph(3’)* | F: GCCGATGTGGATTGCGAAAA | 292 bp | 58℃ | ([Tian et al., 2019](#_ENREF_3)) |
|  | R: GCTTGATCCCCAGTAAGTCA |  |  |  |
| *Sul1* | F: CATTGCCTGGTTGCTTCAT | 238 bp | 56℃ |  |
|  | R: ATCCGACTCGCAGCATTT |  |  |  |
| *Sul2* | F: CATCATTTTCGGCATCGTC | 793 bp | 56℃ |  |
|  | R: TCTTGCGGTTTCTTTCAGC |  |  |  |
| *Sul3* | F: AGATGTGATTGATTTGGGAGC | 443 bp | 56℃ |  |
|  | R: TAGTTGTTTCTGGATTAGAGCCT |  |  |  |

**References**

Frank, J.A., Reich, C.I., Sharma, S., Weisbaum, J.S., Wilson, B.A., and Olsen, G.J. (2008). Critical evaluation of two primers commonly used for amplification of bacterial 16S rRNA genes. *Appl Environ Microbiol* 74**,** 2461-2470.

Jacobs, M.R., Bajaksouzian, S., and Appelbaum, P.C. (2003). Telithromycin post-antibiotic and post-antibiotic sub-MIC effects for 10 Gram-positive cocci. *J Antimicrob Chemother* 52**,** 809-812.

Tian, X.Y., Zheng, N., Han, R.W., Ho, H., Wang, J., Wang, Y.T., Wang, S.Q., Li, H.G., Liu, H.W., and Yu, Z.N. (2019). Antimicrobial resistance and virulence genes of *Streptococcus* isolated from dairy cows with mastitis in China. *Microb Pathog* 131**,** 33-39.

Zhang, C., Zhang, Z., Song, L., Fan, X., Wen, F., Xu, S., and Ning, Y. (2015). Antimicrobial Resistance Profile and Genotypic Characteristics of *Streptococcus suis* Capsular Type 2 Isolated from Clinical Carrier Sows and Diseased Pigs in China. *Biomed Res Int* 2015**,** 284303.
