# Supplementary material for: Prevalence and antimicrobial resistance of Streptococcus suis isolated from local pig breeds in Jiangxi Province, China
Source: Front Vet Sci. 2025 Aug 4;12:1582461. doi: 10.3389/fvets.2025.1582461 (PMC12358291; doi:10.3389/fvets.2025.1582461)
Supplement: Supplementary file 2 [file Table_2.DOCX]

Table S2**.** Specific characteristics for each of the antibiotics used in this study.

| Antimicrobials | Drug content (μg) | Diameter of antibacterial zone (mm) | | |
| --- | --- | --- | --- | --- |
|  |  | Resistant | Intermediate | Susceptible |
| Amoxicillin | 20 | – | – | ≥ 28 |
| Ampicillin | 10 | ≤ 18 | 19-25 | ≥ 26 |
| Ceftiofur | 30 | – | – | ≥ 28 |
| Doxycycline | 30 | ≤ 12 | 13-15 | ≥ 16 |
| Chlortetracychine | 30 | – | – | ≥ 19 |
| Florfenicol | 30 | ≤ 17 | 18-20 | ≥ 21 |
| Erythromycin | 15 | ≤ 15 | 16-20 | ≥ 21 |
| Tilmicosin | 15 | – | – | ≥ 19 |
| Colistin sulfate | 10 | – | – | ≥ 11 |
| Sulfadiazine | 300 | – | – | ≥ 20 |
| Enrofloxacin | 10 | – | – | ≥ 22 |
| Ciprofloxacin | 5 | ≤ 15 | 16-20 | ≥ 21 |
| Tiamulin | 30 | – | – | ≥ 25 |
| Neomycin | 30 | ≤ 12 | 13-16 | ≥ 17 |
| Spectinomycin | 100 | ≤ 14 | 15-17 | ≥ 18 |
| Gentamicin | 10 | ≤ 12 | 13-14 | ≥ 15 |
| Kanamycin | 30 | ≤ 13 | 14-17 | ≥ 18 |
| Apramycin | 15 | – | – | ≥ 17 |
| Imipenem | 10 | ≤ 13 | 14-15 | ≥ 16 |
| Meropenem | 10 | – | – | ≥ 18 |
