# Supplementary material for: Prevalence and antimicrobial resistance of Streptococcus suis isolated from local pig breeds in Jiangxi Province, China
Source: Front Vet Sci. 2025 Aug 4;12:1582461. doi: 10.3389/fvets.2025.1582461 (PMC12358291; doi:10.3389/fvets.2025.1582461)
Supplement: Supplementary file 4 [file Table_4.DOCX]

Table S4. Antimicrobial susceptibility testing results of three carbapenem-resistant bacterial strains. S, susceptible; I, intermediate; R, resistant.

| Strain | Disk diffusion method | | Minimum inhibitory concentration (MIC) | |
| --- | --- | --- | --- | --- |
|  | Imipenem^a^ | Meropenem^b^ | Imipenem^c^ | Meropenem^d^ |
| S050 | 21 mm (S) | 15 mm (R) | 0.75 μg/mL (I) | 2 μg/mL (R) |
| S111 | 0 mm (R) | 0 mm (R) | ＞32 μg/mL (R) | ＞32 μg/mL (R) |
| S145 | 12 mm (R) | 15 mm (R) | 1 μg/mL (R) | 3 μg/mL (R) |

^a^The breakpoints: susceptible (≤ 13 mm), intermediate (14-15 mm), and resistant (≥ 16 mm).

^b^The breakpoints: susceptible (﹤18 μg/mL) and resistant (≥18 mm).

^c^The breakpoints: susceptible (≤ 0.12 μg/mL), intermediate (0.25-0.5 μg/mL), and resistant (≥ 1 μg/mL).

^d^The breakpoints: susceptible (≤ 0.25 μg/mL), intermediate (0.5 μg/mL), and resistant (≥ 1 μg/mL).
